# Alteration of functional connectivity network in population of objectively-defined subtle cognitive decline

**DOI:** 10.1093/braincomms/fcae033

**Published:** 2024-02-09

**Authors:** Xinyi Zhang, Qingze Zeng, Yanbo Wang, Yu Jin, Tiantian Qiu, Kaicheng Li, Xiao Luo, Shuyue Wang, Xiaopei Xu, Xiaocao Liu, Shuai Zhao, Zheyu Li, Luwei Hong, Jixuan Li, Siyan Zhong, Tianyi Zhang, Peiyu Huang, Baorong Zhang, Minming Zhang, Yanxing Chen

**Affiliations:** 1 Department of Neurology, The Second Affiliated Hospital of Zhejiang University School of Medicine, 310009, Hangzhou, China; Department of Radiology, The Second Affiliated Hospital of Zhejiang University School of Medicine, 310009, Hangzhou, China; 1 Department of Neurology, The Second Affiliated Hospital of Zhejiang University School of Medicine, 310009, Hangzhou, China; 1 Department of Neurology, The Second Affiliated Hospital of Zhejiang University School of Medicine, 310009, Hangzhou, China; Department of Radiology, Linyi People’s Hospital, 276003, Linyi, China; Department of Radiology, The Second Affiliated Hospital of Zhejiang University School of Medicine, 310009, Hangzhou, China; Department of Radiology, The Second Affiliated Hospital of Zhejiang University School of Medicine, 310009, Hangzhou, China; Department of Radiology, The Second Affiliated Hospital of Zhejiang University School of Medicine, 310009, Hangzhou, China; Department of Radiology, The Second Affiliated Hospital of Zhejiang University School of Medicine, 310009, Hangzhou, China; Department of Radiology, The Second Affiliated Hospital of Zhejiang University School of Medicine, 310009, Hangzhou, China; 1 Department of Neurology, The Second Affiliated Hospital of Zhejiang University School of Medicine, 310009, Hangzhou, China; 1 Department of Neurology, The Second Affiliated Hospital of Zhejiang University School of Medicine, 310009, Hangzhou, China; Department of Radiology, The Second Affiliated Hospital of Zhejiang University School of Medicine, 310009, Hangzhou, China; Department of Radiology, The Second Affiliated Hospital of Zhejiang University School of Medicine, 310009, Hangzhou, China; 1 Department of Neurology, The Second Affiliated Hospital of Zhejiang University School of Medicine, 310009, Hangzhou, China; Department of Neurology, The First Affiliated Hospital of Zhejiang University School of Medicine, 310003, Hangzhou, China; Department of Radiology, The Second Affiliated Hospital of Zhejiang University School of Medicine, 310009, Hangzhou, China; 1 Department of Neurology, The Second Affiliated Hospital of Zhejiang University School of Medicine, 310009, Hangzhou, China; Department of Radiology, The Second Affiliated Hospital of Zhejiang University School of Medicine, 310009, Hangzhou, China; 1 Department of Neurology, The Second Affiliated Hospital of Zhejiang University School of Medicine, 310009, Hangzhou, China

**Keywords:** Alzheimer’s disease, objectively-defined subtle cognitive decline, degree centrality, eigenvector centrality, functional connectivity

## Abstract

The objectively-defined subtle cognitive decline individuals had higher progression rates of cognitive decline and pathological deposition than healthy elderly, indicating a higher risk of progressing to Alzheimer’s disease. However, little is known about the brain functional alterations during this stage. Thus, we aimed to investigate the functional network patterns in objectively-defined subtle cognitive decline cohort. Forty-two cognitive normal, 29 objectively-defined subtle cognitive decline and 55 mild cognitive impairment subjects were included based on neuropsychological measures from the Alzheimer’s disease Neuroimaging Initiative dataset. Thirty cognitive normal, 22 objectively-defined subtle cognitive declines and 48 mild cognitive impairment had longitudinal MRI data. The degree centrality and eigenvector centrality for each participant were calculated by using resting-state functional MRI. For cross-sectional data, analysis of covariance was performed to detect between-group differences in degree centrality and eigenvector centrality after controlling age, sex and education. For longitudinal data, repeated measurement analysis of covariance was used for comparing the alterations during follow-up period among three groups. In order to classify the clinical significance, we correlated degree centrality and eigenvector centrality values to Alzheimer’s disease biomarkers and cognitive function. The results of analysis of covariance showed significant between-group differences in eigenvector centrality and degree centrality in left superior temporal gyrus and left precuneus, respectively. Across groups, the eigenvector centrality value of left superior temporal gyrus was positively related to recognition scores in auditory verbal learning test, whereas the degree centrality value of left precuneus was positively associated with mini-mental state examination total score. For longitudinal data, the results of repeated measurement analysis of covariance indicated objectively-defined subtle cognitive decline group had the highest declined rate of both eigenvector centrality and degree centrality values than other groups. Our study showed an increased brain functional connectivity in objectively-defined subtle cognitive decline individuals at both local and global level, which were associated with Alzheimer’s disease pathology and neuropsychological assessment. Moreover, we also observed a faster declined rate of functional network matrix in objectively-defined subtle cognitive decline individuals during the follow-ups.

## Teaser

Zhang *et al*. report that the objectively-defined subtle cognitive decline individuals showed an increased brain functional connectivity at both local and global level, which were associated with Alzheimer’s disease pathology and neuropsychological assessment. Moreover, they also observed a faster declined rate of functional network matrix in the follow-up cohort of this population.

## Graphical Abstract

**Graphical abstract:**
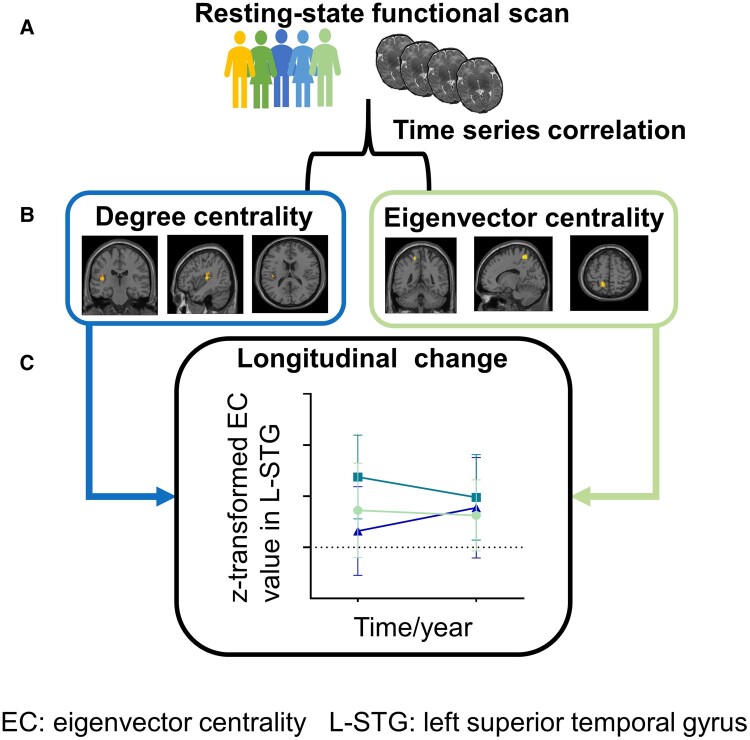

See Negro and Opazo (https://doi.org/10.1093/braincomms/fcae050) for a scientific commentary on this article.

## Introduction

Alzheimer’s disease is the most common form of dementia, marked by progressive loss of memory and other cognitive functions.^1^ Given the absence of disease-modifying therapy for Alzheimer’s disease, it becomes crucial to identify patients at high risk during the preclinical stage for early interventions. In this context, the concept of objectively-defined subtle cognitive decline (Obj-SCD) has been introduced to precisely capture the subtle cognitive decline occurring before clinical onset.^2,3^ The Obj-SCD operationally distinguishes individuals who have subtle cognitive impairment during the preclinical stage based on specific neuropsychological measures.^2,3^ Recent studies have shown that the Obj-SCD cohort exhibits higher cognitive decline rates and a more rapid accumulation of amyloid-β (Aβ) protein burden compared to healthy controls,^3,4^ thus highlighting its elevated risk of progressing to Alzheimer’s disease. Despite these advances, the alterations in cerebral structure or function during the Obj-SCD stage remain largely unexplored.

Resting-state functional magnetic resonance imaging (rs-fMRI) is a powerful and non-invasive tool for exploring brain functional changes *in vivo*, and it has been widely used in neurodegenerative diseases such as Alzheimer’s disease, Parkinson’s disease and Huntington’s disease.^5-10^ Previous research has highlighted that the pathological deposition associated with Alzheimer’s disease can impair synaptic communication, thereby leading to the disruption of brain networks.^11-13^ In this framework, viewing the whole brain as an interconnected network, graph theoretical centrality metrics can be employed to quantify the nodal importance and network alterations in specific brain regions. Degree Centrality (DC) and Eigenvector Centrality (EC) stand as key parameters for assessing voxel-wise connectivity matrices across the entire brain, with EC and DC reflecting global and local metrics, respectively.^14,15^ Prior studies have shown that individuals within the Alzheimer’s disease spectrum exhibit brain network alterations, as measured by EC or DC values, particularly in Alzheimer’s disease -vulnerable regions like the hippocampus,^16,17^ precuneus^17,18^ and superior temporal gyrus (STG).^19^ Since Obj-SCD marks the early cognitive decline in the progression of Alzheimer’s disease, exploring the brain network alterations in this population is especially important. However, research focused on this area remains scarce. To cover this gap, we employed DC and EC to investigate the brain network changes at Obj-SCD stage.

We aimed to explore the alterations in the intrinsic functional network and its relationship with Alzheimer’s disease pathologies and cognitive function in Obj-SCD individuals. Building on the existing literature that posits Obj-SCD as a very early stage of Alzheimer’s disease, we hypothesized that Obj-SCD individuals may exhibit higher functional network indexes compared to patients with mild cognitive impairment (MCI).

## Materials and methods

### Study population

Data used in the preparation of this article were obtained from the Alzheimer’s disease Neuroimaging Initiative (ADNI) database (http://adni.loni.usc.edu/). Ethical approval was obtained by the ADNI investigators, all participants provided written informed consent (further information about the inclusion/exclusion criteria may be found at www.adni-info.org). In this study, we included 132 participants [46 cognitive normal (CN), 29 Obj-SCD and 57 MCI] who completed fMRI scan and neuropsychological assessment, and 100 participants (30 CN, 22 Obj-SCD and 48 MCI) have the longitudinal rs-fMRI data at 6 months after baseline. The classification criteria of Obj-SCD and MCI are described in the *Neuropsychological assessment* section.

### Neuropsychological assessment

For determining MCI classification, we used six neuropsychological variables as total test scores which involve three different cognitive domains including memory domains [Rey Auditory Verbal Learning Test (AVLT) delayed free recall correct responses and AVLT recognition discrimination (hits minus false positives)], language domains (30-item Boston Naming Test [BNT] total correct and Animal Fluency Test [AFT] total score), and attention/executive functioning domains (Trail Making Test Parts A [TMT-A] and B [TMT-B] times to completion).

For the Obj-SCD classification, additional three process scores derived from the AVLT were used in the current study, including learning slope [(List A Trial 5—List A Trial 1)/5], retroactive interference (List A Trial 6/List A Trial 5), and total intrusion errors (total number of extra-list intrusion errors across all recall trials).

### Cognitive classifications

One thousand three hundred and eighty non-demented ADNI participants who completed a baseline neuropsychological assessment were considered for analyses. To begin with, we used a sample of 239 CN participants in ADNI who did not progress to MCI with at least 4 years of follow-up (range 4–15 years; mean 7.18 years) to identify the robust normative control group. Next, we ran the regressions on this robust normal control group. For each neuropsychological test (including the neuropsychological process scores), we regressed the test score on age, education and sex in order to get the regression weights. Subsequently, for all participants, we calculated the predicted test score for each test using these regression b-weights derived from the normative control group. Then, participant z-scores were then calculated based on the discrepancy between the observed and predicted scores and divided by the test-specific control group’s regression model’s standard error of the estimate as shown in the following formula:


$$z\text{-}\mathrm{scores}=\frac{\mathrm{Observed}\;\mathrm{Test}\;\mathrm{Score}\;\text{–}\;\mathrm{Predicted}\;\mathrm{Test}\;\mathrm{Score}}{\mathrm{Standard}\;\mathrm{Error}\;\mathrm{of}\;\mathrm{the}\;\mathrm{Estimate}}$$

Next, we applied a threshold of >1 SD (i.e. z-score < −1) ^2^below the mean to categorize impairment on a given test.

Participants were considered MCI if they did not have dementia and performed >1 SD below the age-/education-/sex-adjusted mean (i.e. z-score < −1) on (i) two neuropsychological measures within the same cognitive domain, or (ii) at least one measure across all three sampled cognitive domains. Within ADNI, the six neuropsychological total test scores mentioned earlier were utilized in establishing the MCI criteria.^2,20,21^

Consequently, the remaining participants were considered to have Obj-SCD if they scored >1 SD below the age-/education-/sex-adjusted mean under any of the following conditions: (i) one impaired total test score in two different cognitive domains (memory, language and attention/executive), or (ii) two impaired neuropsychological process scores from the AVLT, or (iii) one impaired total test score and one impaired process score.

### Cerebrospinal fluid biomarkers analysis

Cerebrospinal fluid (CSF) biomarkers included amyloid-beta 42 (Aβ_42_), total tau (t-tau), and phosphorylated tau at position 181 (p-tau_181_), were all measured by multiplex xMAP Luminex platform (Luminex) with Innogenetics (INNOBIA AlzBio3) immunoassay kit-based reagents.^22^ Notably, not all subjects had CSF samples since lumbar puncture is an invasive procedure. In the current study, 121 subjects have available CSF biomarker data (41 CN, 28 Obj-SCD and 52 MCI). The recommended threshold of Aβ_42_ in CSF <192 ng/L was used to determine abnormal amyloid deposition (A+).^22,23^ We combined the above data and further divided participants into A– CN (*N* = 24), A + Obj-SCD (*N* = 19) and A + MCI (*N* = 34).

### Plasma biomarkers analysis

Blood sampling and processing were conducted by the ADNI protocol. Plasma neurofilament light chain (NfL) concentration was analyzed by the Single Molecule Array (SiMoA) technique as the previous papers.^24^

### MRI acquisition and preprocessing

All participants were scanned using a 3.0-Tesla MRI scanner. The rs-fMRI scans were obtained using an echo-planar imaging (EPI) sequence with the following parameters:140 time points; repetition time (TR) = 3000 ms; echo time (TE) = 30 ms; flip angle = 80°; number of slices = 48; slice thickness = 3.3 mm; spatial resolution = 3.31 × 3.31 × 3.31 mm^3^; matrix = 64 × 64. Here, one CN subject was discarded due to poor image quality.

The rs-fMRI data were preprocessed by using the Data Processing and Analysis for Brain Imaging (DPABI Version 6.0)^25^ toolbox with Statistical Parametric Mapping 12 (SPM12, http://www.fil.ion.ucl.ac.uk/spm/) on the MATLAB platform (MathWorks, Natick, MA, USA). First, the first 10 time points were discarded for the signal equilibrium and subject’s adaptation to the scanning noise. Second, the remaining 130 images were corrected for timing differences and head motion (Friston 24 parameter). The parameter of head motion of each subject was calculated by using frame-wise displacement (FD, from JD Power’s method). Two MCI patients and three CN subjects were excluded due to their excessive head movement (more than 3 mm maximum displacement in any of the *x-*, *y-* or *z*-directions or 3° of any angular motion). Third, the rs-fMRI images for each subject were spatially normalized to the EPI template in Montreal Neurological Institute (MNI) standard space and then re-sampled into 3 × 3 × 3 mm^3^ cubic voxels. Fourth, we removed linear trends and regressed out covariates, including Friston 24 head motion parameters, and averaged signals of white matter (WM) and CSF. Finally, we conducted a filter procedure (0.01–0.1 Hz) to remove the bias from the high-frequency physiological noise and the low-frequency drift.

### Centrality metrics

After preprocessing, for each subject, Pearson’s correlations in the time series between each voxel with every other voxel were calculated to produce a functional connectivity matrix within the gray matter mask which was generated by setting a threshold of 0.3 on the gray matter probability. Then, DC and EC were calculated in a voxel-wise manner to assess the local and global functional connectivity of the brain networks respectively. In brief, voxel-based DC was computed with DPARSFA (version 5.2), by counting the number of voxels it was correlated to at a threshold of r ≥ 0.25.^26-29^ At the same time, EC was calculated by counting the weighted number of correlations with the Fast Eigenvector Centrality Mapping (fECM) toolbox (https://www.github.com/amwink/bias/tree/master/matlab/fastECM).^26,30^ Subsequently, DC and EC maps across all participants underwent Fisher’s Z transformation and smoothing with a Gaussian kernel of 6 × 6 × 6 mm^3^ full widths at half maximum.

### Ethical standards

All procedures performed in studies involving human participants were in accordance with the ethical standards of the institutional and national research committee and with the 1964 Helsinki Declaration and its later amendments or comparable ethical standards.

Written informed consent was obtained from all participants authorized representatives, and the study partners before any protocol-specific procedures were carried out in the ADNI study. More details at http://www.adni-info.org.

### Statistical analyses

The demographic and clinical characteristics were analyzed using SPSS (IBM SPSS Statistics, Version 25.0). We used the analysis of variance (ANOVA) to compare the continuous data among three groups; subsequently, we performed the *post hoc* two-sample *t*-test. The Chi-square test was used for categorical data assessment. Results with *P* < 0.05 were considered statistically significant.

The comparison of imaging measures was performed by using DPABI toolbox. We used analysis of covariance (ANCOVA) to detect the difference in DC and EC values among CN, Obj-SCD and MCI controlling for the age, gender, education and mean FD. The Gaussian random field (GRF) correction was used to correct for multiple comparisons. The statistical threshold was set at voxel-level *P* < 0.005 with a cluster-level *P* < 0.05 (two-tailed) in DPABI. Then, the clusters with significant between-group differences were defined as regions of interest (ROIs), and the mean DC and EC values were extracted in these ROIs. Next, we performed a *post hoc* analysis between each pair of groups. Subsequently, we correlated these neuroimaging metrics with age, gender distribution, years of education, cognitive assessments and CSF biomarkers. *Post hoc t-*tests and correlation analyses were corrected for multiple comparisons by least significant difference (LSD).

In addition, in order to improve the reliability of our study, we repeated our analysis in participants grouped by both cognitive and amyloid burden.

For longitudinal data analysis, we extracted the DC and EC values in statistically significant ROIs at baseline, and then we performed repeated measure ANOVA to illustrate the change pattern of the DC and EC among the participants with the longitudinal rs-fMRI data.

## Result

### Cross-sectional cohort

#### Participant characteristic

Table 1 shows the demographic and clinical characteristics of participants by cognitive status (CN: *N* = 42, Obj-SCD: *N* = 29, MCI: *N* = 55). There were no significant differences in age, gender distribution, years of education or number of apolipoprotein E (*AOPE*) ɛ4 Carrier among the three groups. There were significant differences in the performance of neuropsychological tests except for total intrusion errors in all samples. Significant differences were also observed in the levels of NfL in plasma, but not of Aβ_42_, p-tau_181_ and t-tau in CSF.

**Table 1:** Sociodemographic and clinical characteristics of CN, Obj-SCD and MCI subjects

	CN (*N* = 42)	Obj-SCD (*N* = 29)	MCI (*N* = 55)	*F/X^2^*	*P*
Age, year	71.29 ±6.85	71.97 ±6.47	73.58 ±6.66	1.489	0.230
Gender (F/M), *N*	24/18	14/15	27/28	0.784	0.676
AOPE ɛ4 Carrier, *N* (42/28/55)	16(38.10%)	13(46.43%)^a^	20(36.36%)	0.821	0.663
Education, year	16.36 ±2.56	16.59 ±2.73	15.76 ±2.49	1.178	0.311
MMSE	28.74 ±1.21	28.59 ±1.59	27.85 ±1.79^b,c^	4312	0.015
AVLT 30 min delayed recall	8.36 ±2.79	5.21 ±3.08^d^	3.15 ±3.34^b,c^	33.551	<0.001
AVLT-recognition	12.76 ±1.85	11.79 ±2.11	8.36 ±3.59^b,c^	32.870	<0.001
AFL	22.05 ±4.03	20.59 ±4.08	17.29 ±4.45^b,c^	15.985	<0.001
BNT	28.42 ±1.44	27.72 ±1.81	26.60 ±3.66^b^	6.142	0.003
TMT-A	29.48 ±7.71	27.72 ±1.81	44.91 ±17.26^b,c^	20.492	<0.001
TMT-B	67.00 ±17.75	89.07 ±40.75	125.96 ±68.46^b,c^	16.860	<0.001
Learning slope	1.33 ±0.39	0.91 ±0.45^d^	0.74 ±0.46^b^	22.394	<0.001
Retroactive interference	0.80 ±0.17	0.69 ±0.27^d^	0.59 ±0.24^b^	10.961	<0.001
Total intrusion errors	2.24 ±2.38	3.82 ±2.90^d^	3.51 ±3.49^b^	3.023	0.052
CSF Aβ_42_, pg/mL (37/28/50)	195.84 ±54.64	170.81 ±48.35	182.35 ±56.57	1.743	0.180
CSF p-tau_181_, pg/mL (37/28/50)	37.71 ±21.99	42.98 ±18.05	41.80 ±26.97	0.485	0.617
CSF t-tau, (pg/mL) (37/28/48)	77.37 ±50.06	86.88 ±47.00	81.16 ±43.81^e^	0.331	0.719
Plasma NfL, (pg/mL) (41/26/53)	31.51 ±12.91	31.90 ±9.69	39.89 ±16.46^b,c^	5.073	0.008

Data are presented as means ±standard deviations.

^a^There was one missing values in the Obj-SCD group.

^b^Statistical significance, *P* < 0.05 (after LSD correction), compared to CN.

^c^Statistical significance, *P* < 0.05 (after LSD correction), compared to Obj-SCD.

Notably: The CSF data in Table 1 only represents the subjects who had CSF sample.

^d^Statistical significance, *P* < 0.05 (after LSD correction), compared to CN.

^e^There were two missing values in the MCI group.

Abbreviation: CN, cognitive normal; Obj-SCD, objectively-defined subtle cognitive decline; MCI, mild cognitive impairment; MMSE, Mini-Mental State Examination; AVLT 30 min delayed recall, Auditory Verbal Learning Test 30 min delayed free recall; AVLT-recognition, Auditory Verbal Learning Test recognition discrimination; AFL, Animal Fluency total score; BNT, Boston Naming Test; TMT-A, Trail Making Test A; TMT-B, Trail Making Test B; t-tau, total tau; p-tau, phosphorylated tau; Aβ_42_, amyloid-beta_42._; NfL, neurofilament light chain.

#### Between-group differences of centrality matrix

**Figure 1:**
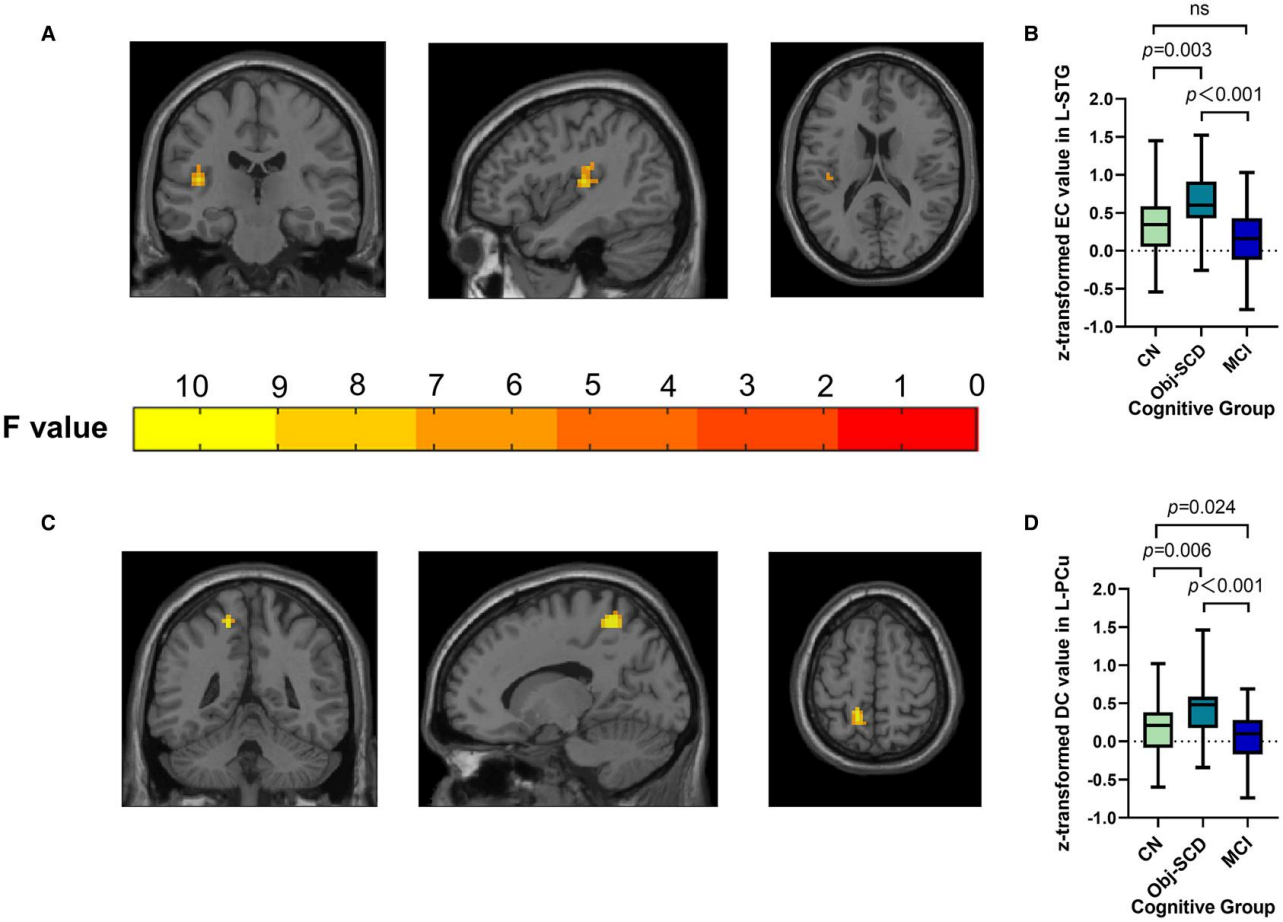
**Shows the between-group differences of centrality matrix across three groups.** (**A** and **B**). Obj-SCD individuals showed higher EC value in L-STG. (**C** and **D**). Obj-SCD individuals showed higher DC value in L-PCu. The imaging results were obtained by ANCOVA analysis adjusted with mean age, gender, education and mean FD (*P* < 0.005, cluster level < 0.05, two-tailed, GRF correction), the difference of EC and DC values were obtained by using post hoc *t-* tests after controlling for age, gender, education, and mean FD [*P* < 0.05, two-tailed, least significance difference (LSD) correction]. Abbreviations: Obj-SCD, objectively-defined subtle cognitive decline; EC, eigenvector centrality; DC, degree centrality; L-STG, left superior temporal gyrus; L-PCu, left precuneus.

Regarding image analysis, the results showed the between-group differences of EC in left superior temporal gyrus (L-STG). In addition, we found significant differences in DC value in left precuneus (L-PCu) among the three groups. The *post hoc* analyses showed that the participants in Obj-SCD group had higher EC values in L-STG and higher DC values in L-PCu than CN and MCI subjects (Fig. 1). Because *APOE* is an important risk gene for Alzheimer’s disease, we also considered *APOE* genotype as a covariate in statistical analyses, the result showed that brain regions with EC and DC differences remained unchanged after APOE genotype was added to the covariate (Supplementary Fig. 1).

#### Correlation of centrality matrix with neuropsychological performance and CSF biomarkers

**Figure 2:**
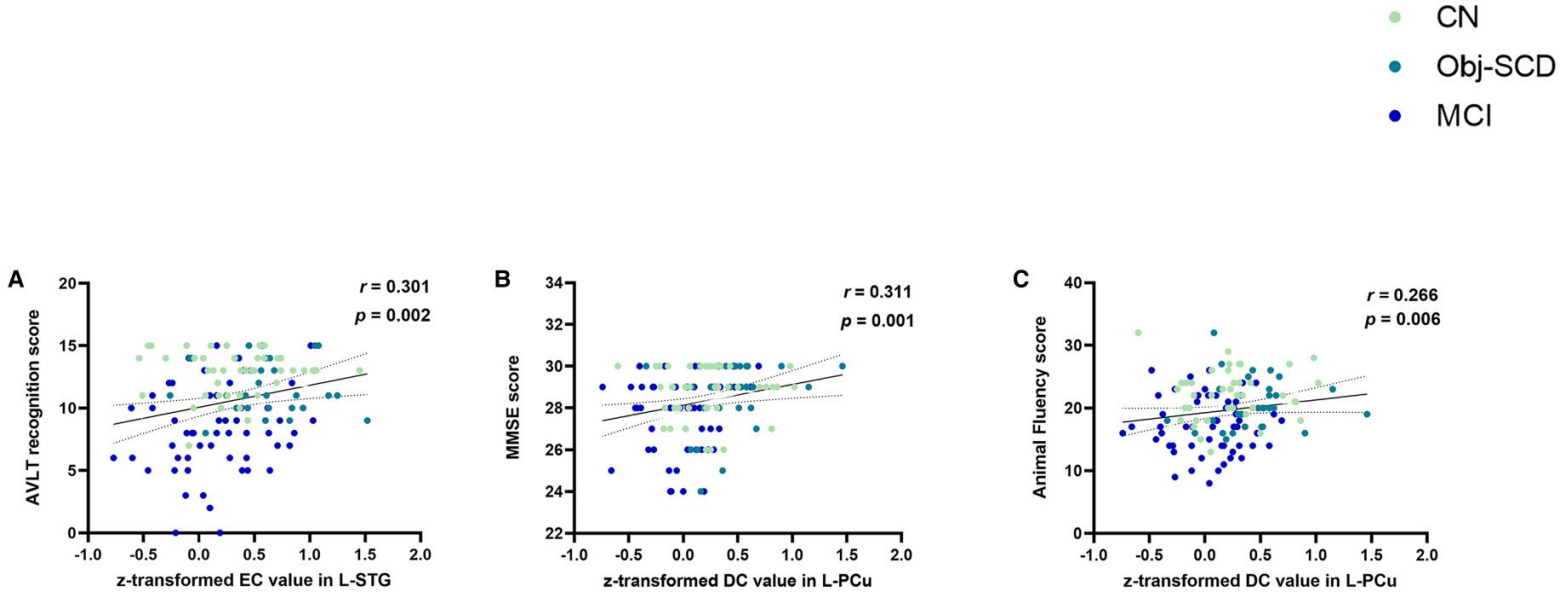
**Shows the association between centrality matrix and neuropathological metrics across groups.** (**A**) EC value of L-STG related with AVLT recognition scores (*r* = 0.301, *P* = 0.002); (**B**) DC value of L-PCu related with MMSE scores (*r* = 0.311, *P* = 0.001); (**C**) DC value of L-PCu related with AFT total scores (*r* = 0.266, *P* = 0.006). The results were obtained by using partial correlation analyses. The scatter plot diagram displays the 95% confidence band of the best-fit line. Abbreviations: EC, eigenvector centrality; DC, degree centrality; L-STG, left superior temporal gyrus; L-PCu, left precuneus; AVLT-recognition, Auditory Verbal Learning Test recognition discrimination.

Across groups, the EC value of L-STG was positively related to AVLT recognition scores (*r* = 0.301*, P* = 0.002) (Fig. 2A). Meanwhile, the DC value of L-PCu across three groups was positively related to the sores of Mini-mental State Examination (MMSE) and Animal Fluency total score (*r* = 0.311, *P* = 0.001; *r = 0.266, P* = 0.006, respectively) (Fig. 2B and C). However, neither EC value of L-STG nor DC value of L-PCu correlated with the CSF biomarkers.

#### Supplementary analyses in sub-sample restricting amyloid status

**Figure 3:**
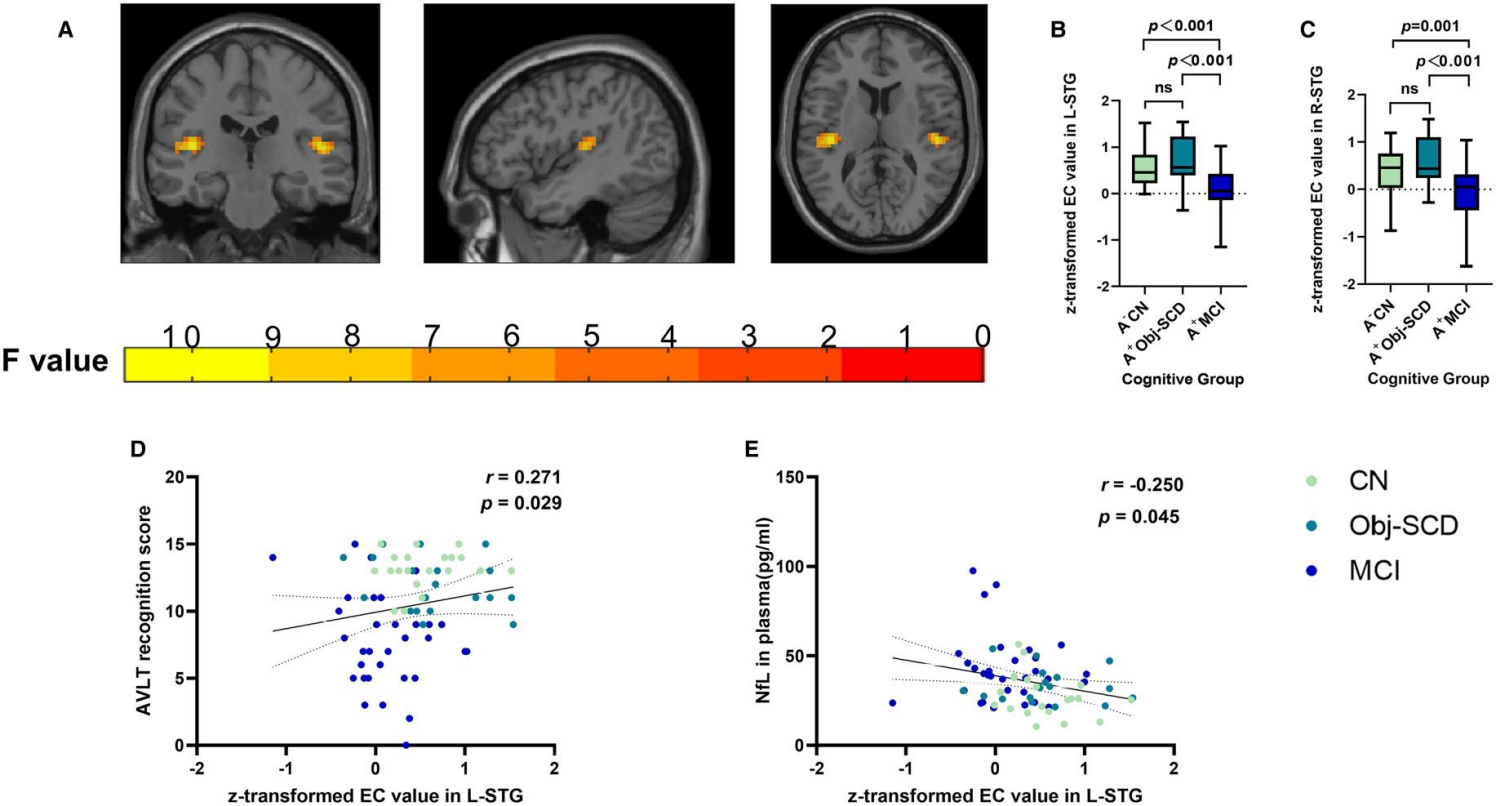
**Shows the between-group differences of EC and association between EC and neuropathological and neuropsychological results across groups in sub-sample restricting amyloid status.** (**A**–**C**). A + Obj-SCD individuals showed higher EC value in B-STG among three groups. The imaging results were obtained by ANCOVA adjusted with mean age, gender, education and mean FD (*P* < 0.005, cluster level <0.05, two-tailed, GRF correction), the difference of EC and DC values were obtained by using post hoc t-tests after controlling for age, gender, education, and mean FD [*P* < 0.05, two-tailed, least significance difference (LSD) correction]. (**D**) EC value of L-STG correlated with AVLT recognition scores (*r* = 0.271, *P* = 0.029); (**E**) EC value of L-STG negatively correlated with the concentration of NfL in plasma (*r* = −0.250, *P* = 0.045). Results of correlation were obtained by analysis of partial correlation analysis. The scatter plot diagram displays the 95% confidence band of the best-fit line. Abbreviations: Obj-SCD, objectively-defined subtle cognitive decline; EC, eigenvector centrality; L-STG, left superior temporal gyrus; R-STG, right superior temporal gyrus; AVLT-recognition, Auditory Verbal Learning Test recognition discrimination; NfL, neurofilament light chain.

The repeated analyses in the sub-sample showed significant differences in EC value of bilateral superior temporal gyrus (B-STG) among the three groups. However, there were no differences in DC value. *Post hoc* analyses revealed that the subjects with Obj-SCD had the highest level of EC value in B-STG than other groups (Fig. 3A–C). Across groups, the EC value of L-STG was positively correlated with AVLT recognition scores (*r* = 0.271, *P* = 0.029) and negatively correlated with the concentration of NfL in plasma (*r* = –0.250*, P* = 0.045) (Fig. 3D–E).

### Longitudinal cohort

#### Participant characteristics

Table 2 shows the demographic and clinical characteristics of participants who had longitudinal data of rs-fMRI within 6 months in three groups (CN: *N* = 30, Obj-SCD: *N* = 22 and MCI: *N* = 48). There were no significant differences in age, gender distribution or years of education among the three groups. However, there were significant differences in the performance of all kinds of neuropsychological tests we employed among the three groups. Significant differences in the levels of plasma NfL were also observed among the three groups, but not for Aβ_42_, p-tau_181_ and t-tau.

**Table 2:** Sociodemographic and clinical characteristics of subjects with longitudinal data

	CN (*N* = 30)	Obj-SCD (*N* = 22)	MCI (*N* = 48)	*F/X^2^*	*P*
Age, year	70.55 ±7.51	71.36 ±5.87	73.59 ±6.91	2.005	0.140
Gender (F/M), *N*	16/14	10/12	21/27	0.708	0.702
AOPE ɛ4 carrier, *N* (30/22/48)	12(40%)	10(45.45%)^a^	19(39.58%)	0.233	0.890
Education, year	16.63 ±2.16	16.59 ±2.86	15.58 ±2.47	2.173	0.119
MMSE	28.63 ±1.22	28.36 ±1.73	27.63 ±1.79^b,c^	3.936	0.023
AVLT 30 min delayed recall	8.40 ±2.96	5.09 ±3.25^d^	3.00 ±3.22^b,c^	27.112	<0.001
AVLT-recognition	12.60 ±1.98	12.00 ±2.14	8.02 ±3.47^b,c^	29.464	<0.001
AFL	22.83 ±3.38	20.73 ±4.15	17.00 ±4.48^b,c^	19.737	<0.001
BNT	28.60 ±1.35	27.55 ±1.90	26.60 ±3.52^b^	5.044	0.008
TMT-A	30.00 ±8.23	31.00 ±6.19	45.52 ±16.91^b,c^	17.059	<0.001
TMT-B	66.77 ±17.05	91.86 ±44.02	127.81 ±69.58^b,c^	12.529	<0.001
Learning slope	1.34 ±0.40	0.92 ±0.45^d^	0.71 ±0.43^b^	20.372	<0.001
Retroactive interference	0.79 ±0.17	0.68 ±0.28^d^	0.56 ±0.23^b^	9.531	<0.001
Total intrusion errors	2.03 ±1.96	4.05 ±3.23^d^	3.69 ±3.68^b^	3.387	0.038
CSF Aβ_42_, pg/mL (37/28/50)	194.19 ±55.36	168.85 ±46.57	178.41 ±53.73	1.486	0.232
CSF p-tau_181_, pg/mL (37/28/50)	38.89 ±23.62	45.02 ±19.17	42.14 ±27.83	0.373	0.690
CSF t-tau, pg/mL (37/28/48)	80.22 ±56.75	92.25 ±51.00	81.28 ±46.09^e^	0.427	0.654
Plasma NfL, (pg/mL) (41/26/53)	31.07 ±13.52	31.44 ±8.29	40.11 ±17.28^b,c^	4.344	0.016

Data are presented as means ±standard deviations.

^a^There were one missing values in the Obj-SCD group.

^b^Statistical significance, *P* < 0.05(after LSD correction), compared to CN.

^c^Statistical significance, *P* < 0.05(after LSD correction), compared to Obj-SCD.

Notably: The CSF data in Table 2 only represents the subjects who had CSF sample.

^d^Statistical significance, *P* < 0.05(after LSD correction), compared to CN.

^e^There were two missing values in the MCI group.

Abbreviation: CN, cognitive normal; Obj-SCD, objectively-defined subtle cognitive decline; MCI, mild cognitive impairment; MMSE, Mini-Mental State Examination; AVLT 30 min delayed recall, Auditory Verbal Learning Test 30 min delayed free recall; AVLT-recognition, Auditory Verbal Learning Test recognition discrimination; AFL, Animal Fluency total score; BNT, Boston Naming Test; TMT-A, Trail Making Test A; TMT-B, Trail Making Test B; t-tau, total tau; p-tau, phosphorylated tau; Aβ_42_, amyloid-beta_42._; NfL, neurofilament light chain.

#### Changes of EC in L-STG and DC in L-PCu

**Figure 4:**
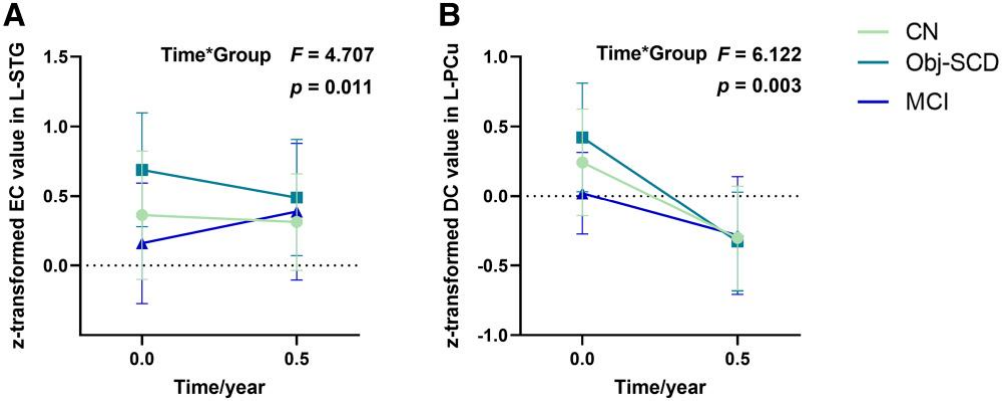
**Shows the change of EC in L-STG and DC in L-PCu.** (**A**) CN and Obj-SCD groups showed higher EC value, while MCI group showed lower EC value in L-STG when compared with their baseline data (CN versus Obj-SCD: *F* = 0.984, *P* = 0.326; CN versus MCI: *F* = 4.328, *P* = 0.041; Obj-SCD versus MCI: *F* = 8.738, *P* = 0.004). (**B**) DC value in L-PCu of three groups dropped compared to baseline data with the Obj-SCD group show the fastest rate of decline (CN versus Obj-SCD: *F* = 1.418, *P* = 0.240; CN versus MCI: *F* = 3.584, *P* = 0.047; Obj-SCD versus MCI: *F* = 11.988, *P* = 0.001). The results were obtained by repeated measures ANCOVA, the difference of EC and DC values were obtained by using post hoc *t-* tests after controlling for age, gender, education, and mean FD [*P* < 0.05, two-tailed, least significance difference (LSD) correction]. Abbreviations: Obj-SCD, objectively-defined subtle cognitive decline; EC, eigenvector centrality; DC, degree centrality; L-STG, left superior temporal gyrus; L-PCu, left precuneus.

Analysis of repeated measurement ANOVA indicated Obj-SCD group had the highest decline rate of EC value in L-STG among the three groups (CN versus Obj-SCD: *F* = 0.984, *P* = 0.326; CN versus MCI: *F* = 4.328, *P* = 0.041; Obj-SCD versus MCI: *F* = 8.738, *P* = 0.004) (Fig. 4A). The DC value of Obj-SCD group in L-PCu showed the fastest decline rate (CN versus Obj-SCD: *F* = 1.418, *P* = 0.240; CN versus MCI: *F* = 3.584, *P* = 0.047; Obj-SCD versus MCI: *F* = 11.988, *P* = 0.001) (Fig. 4B). Besides, after restrict to amyloid status, analysis of repeated measurement ANOVA indicated Obj-SCD group had the highest decline rate of EC value in L-STG among the three groups (CN versus Obj-SCD: *F* = 0.100, *P* = 0.755; CN versus MCI: *F* = 5.187, *P* = 0.029; Obj-SCD versus MCI: *F* = 10.187, *P* = 0.003). However, the DC value in L-PCu show no difference due to the smaller sample size (CN versus Obj-SCD: *F* = 0.607, *P* = 0.444; CN versus MCI: *F* = 0.067, *P* = 0.797; Obj-SCD versus MCI: *F* = 2.731, *P* = 0.107) (Supplementary Fig. 2).

## Discussion

This study investigated the alterations in intrinsic functional network measured by centrality matrix and its underlying mechanisms in Obj-SCD individuals who have a high risk of progression to Alzheimer’s disease. Specifically, we found that the Obj-SCD individuals show higher EC values in L-STG and higher DC values in L-PCu as compared to CN and MCI groups. In addition, the EC value of L-STG was positively related to AVLT recognition scores, while the DC value of L-PCu across three groups was positively related to the sores of MMSE and Animal Fluency total scores. Moreover, most of the results remained unchanged in the EC value in sub-sample restricting amyloid status (A+MCI, A+Obj-SCD and A–CN groups).

We observed obvious differences in EC value in L-STG among three groups at baseline, which was positively related to AVLT recognition scores. EC specifically involves both the quantity and quality of the connections, featuring a global property of centrality.^26,30,31^ There are also other literatures that reported that alterations in EC are involved in the progression of Alzheimer’s disease pathology in non-demented individuals and considered EC as a promising early predictive biomarker.^18,32^ Our repeated analyses in the sub-sample which showed the group differences in EC suggested that initial amyloid pathology affects functional connectivity, thus may reduce the integration among brain regions and exacerbate the deterioration of cognitive function.^18,33^ Superior temporal gyrus (STG) is a brain region that plays a crucial role in memory process and language function. Besides, structural or functional abnormalities such as cortical thickness reductions,^34,35^ amplitude of low-frequency fluctuation signal (ALFF) decline,^36,37^ regional homogeneity (ReHo) alterations,^38,39^ measurable neuronal loss,^40^ and pathology aggregation^41,42^ in STG in patients within Alzheimer’s disease spectrum have been reported by previous findings. In our longitudinal studies, we discovered that the EC value in L-STG displayed a trace of first falling and then rising as the disease progresses. Functional analyses have found that hyperactivity is an early neuronal dysfunction.^43,44^ Thus, we interpreted the falling of EC at Obj-SCD stage as a compensatory response to impairment and speculated the increased activity in MCI stage represented the excessive and ineffective firing of neuronal populations after decompensation.

We found significant differences in DC value of L-PCu at baseline and it was related to the performance of neuropsychological assessment. DC, as local and directly quantifiable centrality measure,^26^ calculating the number of connections at each voxel (node) to the centrality of that voxel (node),^45^ and areas with high DC in the brain means connecting to many distinct areas of the brain at the local level. Early studies have reported abnormalities of local connectivity networks in Alzheimer’s disease spectrum such as individuals with subjective memory complaints (SMC), which holding self-perceived cognitive decline without detectable objective cognitive impairments.^16^ On the other hand, the precuneus is one of brain regions that is vulnerable to Alzheimer’s disease pathology and is the critical part of the default mode network (DMN). The DMN includes a set of brain regions that play an important role in cognition,^46^ and previous studies have demonstrated that dysregulation of functional connectivity in DMN areas is closely associated with disease progression of Alzheimer’s disease.^5,47,48^ In our longitudinal studies, we found that the follow-up DC value of three groups all decreased in comparison with their baseline data, in which the Obj-SCD group showed the most pronounced decline rate. Previous studies have also observed a decline in precuneus brain activity as the disease progresses.^49^ Thus, we speculated the higher DC value in precuneus of Obj-SCD group at baseline might be an early memory impairment within the compensatory range so that the individuals could maintain relatively intact cognitive performance for a period. However, over time, this compensatory function is lost, and a decline in brain activity occurs.

In the present study, we observed higher DC and EC value in Obj-SCD individuals than in CN and MCI individuals at baseline, though in different brain regions. Similarly, regional hyperperfusion has been reported in Obj-SCD individuals.^50^ Animal studies also observed neuronal hyperexcitability during the early stage of Alzheimer’s disease, which shifts subsequently towards hypoexcitability in the advanced stages of disease.^49^ Besides, according to research framework constructed by the National Institute on Aging–Alzheimer’s Association (NIA-AA), which classified the Alzheimer’s disease continuum into six stages by the severity of their clinical symptoms, the A+Obj-SCD individuals are in stage 2, in which the cognitive impairment is milder than those stage 3 A+MCI individuals.^1^ Therefore, Obj-SCD is a stage when mild disease progression exists but remains within the compensation range with relative intact cognition. However, we observed a higher decline rate of DC and EC during the longitudinal period which indicated that the compensatory effect in Obj-SCD is limited.

There exist some limitations in our study. The first one is the relatively small sample size of our study which may undermine the power of data. This may be the reason why the CSF-derived pathology including Aβ_42_, t-tau, and p-tau among three groups did not reach statistical significance. Despite the small sample size, our study provided a new insight for understanding the pathophysiological mechanism of this specific early Alzheimer’s disease stage which bears only mild pathological and cognitive changes. The second one is the fewer remained time points due to the relatively short acquisition (140 time points) and discarding of 10 time points. Although the time point is a bit less, it can provide a preliminary reference for future research, and we hope to improve the study design to make up for this deficiency in subsequent studies. Another limitation is the lack of Positron Emission Computed Tomography (PET) data. Our study used the CSF biomarkers to determine the pathologic burden associated with the formation of amyloid plaque and paired helical filament tau due to the smaller number of subjects who had tau PET data. However, CSF biomarkers are not as precise as PET in measuring amyloid plaque load and pathologic tau deposition in specific vulnerable regions.^1^

## Conclusion

We found enhanced brain functional connectivity of Obj-SCD individuals at both local and global levels in different brain regions which correlated with neuropathological and neuropsychological results. However, we observed that Obj-SCD individuals undergo a faster decline rate during the follow-up period. Our study is helpful in understanding the intrinsic brain functional network changes in Obj-SCD individuals.

## Supplementary Material

fcae033_Supplementary_Data

## Data Availability

The datasets generated and/or analyzed during the current study are available in the ADNI study. More details at http://www.adni-info.org.
