# Supplementary material for: Alteration of functional connectivity network in population of objectively-defined subtle cognitive decline
Source: Brain Commun. 2024 Feb 9;6(1):fcae033. doi: 10.1093/braincomms/fcae033 (PMC10903975; doi:10.1093/braincomms/fcae033)
Supplement: fcae033_Supplementary_Data [file fcae033_supplementary_data.docx]

**SUPPLEMENTARY DATA**

**Table of Contents**

**Supplementary Figure 1:** Shows the between-group differences of centrality matrix across three groups.

**Supplementary Figure 2:** Shows the change of EC in L-STG and DC in L-PCu.


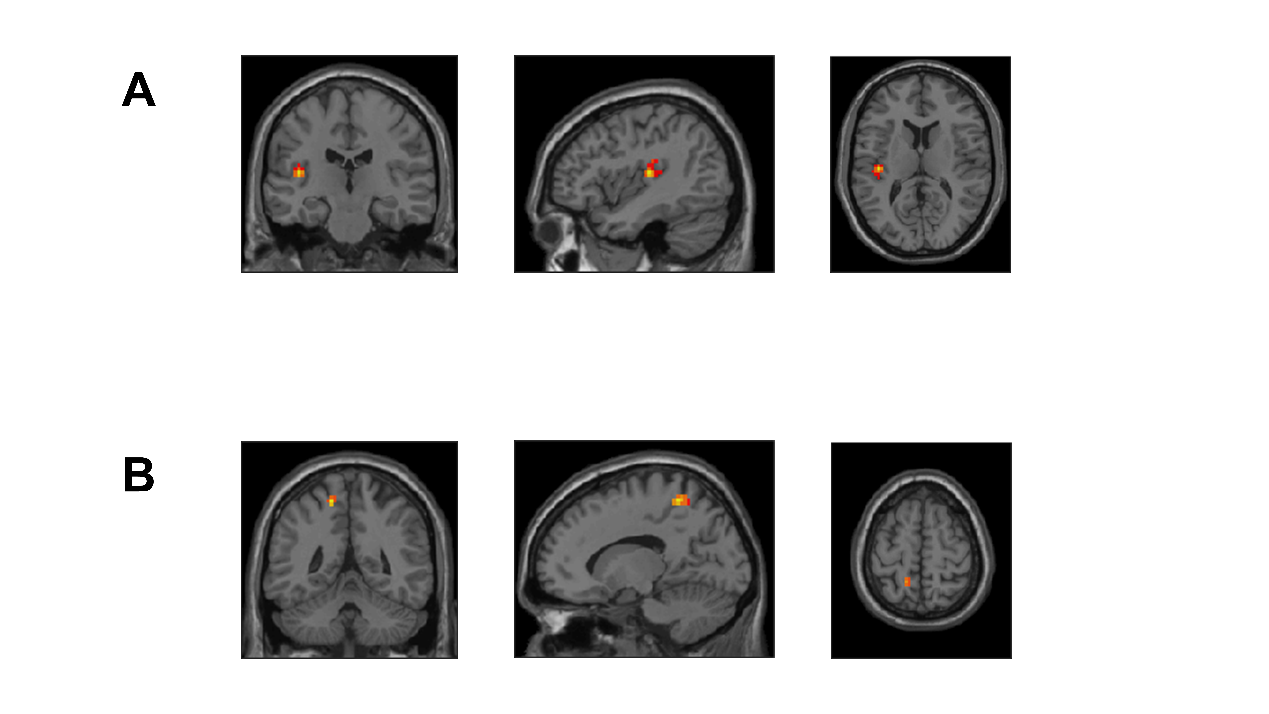


**Supplementary Figure 1:** Shows the between-group differences of centrality matrix across three groups.

A. Brain regions with EC differences remained unchanged after APOE genotype was added to the covariate

B. Brain regions with DC differences remained unchanged after APOE genotype was added to the covariate.

The imaging results were obtained by analysis of covariance (ANCOVA) analysis adjusted with mean age, gender, education, mean frame-wise displacement and APOE genotype [*P* < 0.005, cluster level < 0.05, two-tailed, gaussian random field (GRF) correction].


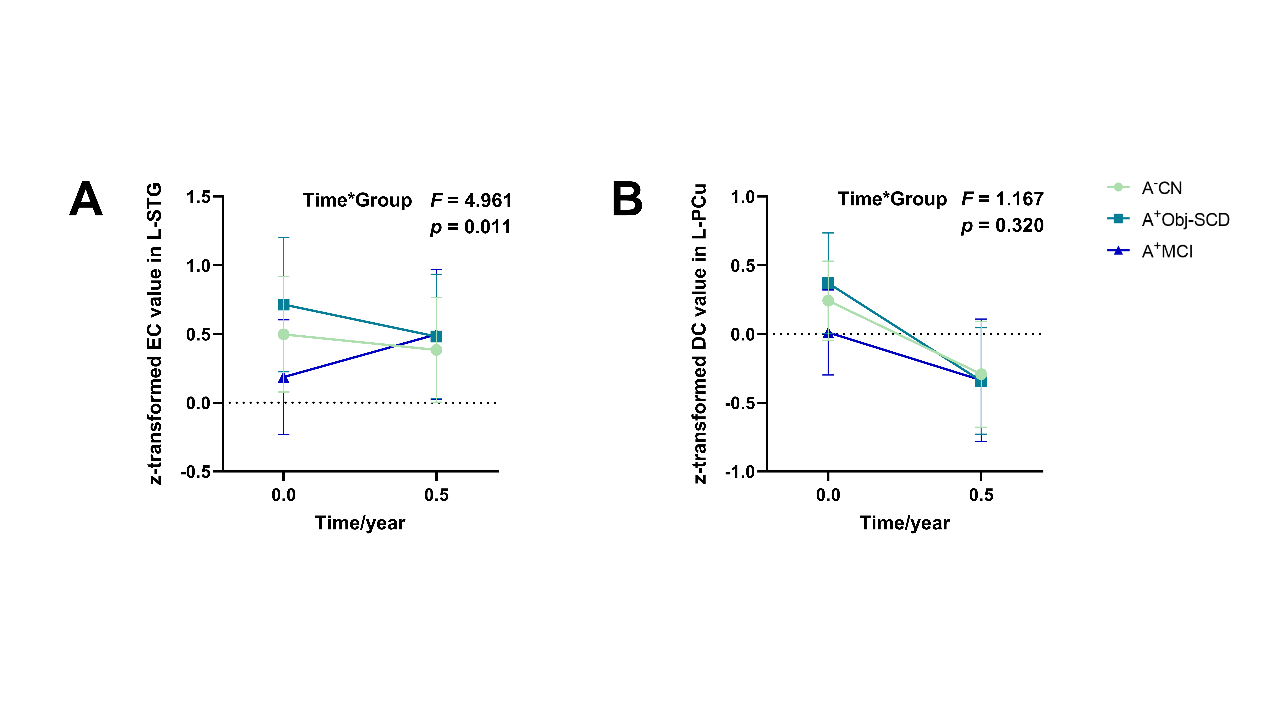


**Supplementary Figure 2:** Shows the change of EC in L-STG and DC in L-PCu.

A. CN and Obj-SCD groups showed higher EC value, while MCI group showed lower EC value in L-STG when compared with their baseline data (CN vs Obj-SCD: *F*=0.100, *p*=0.755; CN vs MCI: *F*=5.187, *p*=0.029; Obj-SCD vs MCI: *F*=10.187, *p*=0.003).

B. DC value in L-PCu of three groups dropped compared to baseline data with the Obj-SCD group show the fastest rate of decline (CN vs Obj-SCD: *F*=0.607, *p*=0.444; CN vs MCI: *F*=0.067, *p*=0.797; Obj-SCD vs MCI: *F*=2.731, *p*=0.107).

The results were obtained by repeated measures ANCOVA.

Abbreviations: Obj-SCD, objectively-defined subtle cognitive decline; EC, eigenvector centrality; DC, degree centrality; L-STG, left superior temporal gyrus; L-PCu, left precuneus.
